# Real-time analysis of arc-induced Long Period Gratings under gamma irradiation

**DOI:** 10.1038/srep43389

**Published:** 2017-03-06

**Authors:** Flavio Esposito, Rajeev Ranjan, Andrei Stăncălie, Dan Sporea, Daniel Neguţ, Nicu Becherescu, Stefania Campopiano, Agostino Iadicicco

**Affiliations:** 1 Department of Engineering, University of Naples “Parthenope”, Centro Direzionale Isola C4, Naples 80143, Italy; 2 National Institute Laser, Plasma and Radiation Physics, Center for Advanced Laser Technologies, 409 Atomiştilor St., Măgurele RO-077125, Romania; 3 “Horia Hulubei” National Institute of Physics and Nuclear Engineering, IRASM Radiation Processing Department, 30 Reactorului St., Măgurele RO-077125, Romania; 4 Apel Laser, 15 Vintila Mihăilescu St., Bucharest RO-060394, Romania

## Abstract

In this paper, we present a comparative experimental and theoretical study on gamma radiation sensitivity of Long Period Gratings (LPGs), fabricated by electric arc discharge technique, as monitored in three single mode optical fibers supplied by different manufacturers. A real-time measurement of LPGs’ wavelength shift was performed until a total dose of 35 kGy was reached, with average dose rate of 0.18 kGy/h, the irradiation being done at room temperature. In one case, a maximum radiation sensitivity of 1.34 nm/kGy was recorded for doses up to 0.5 kGy. Moreover, by combining experimental results with numerical simulations, it was found that changes occurred in the core refractive index of the irradiated optical fibers up to 2.5 ∙ 10^−5^. The increase of the core thermo-optic coefficient up to 1.5 ∙ 10^−8^/°C was observed as well.

Long period gratings (LPGs) are in-fiber devices, which are fabricated by inducing a period perturbation along the optical fiber^1^. The perturbation can be created by acting on the refractive index of silica and/or on the waveguide geometry. Their name is due to the higher period (in the range 100 μm–1 mm) in respect to Fiber Bragg gratings (FBGs)^2^. The transmission spectrum of a LPG is characterized by having discrete attenuation bands, being the result of the power coupling between the fundamental core mode of a single mode fiber and forward propagating cladding modes. These bands are positioned at the wavelengths *λ*_*res,i*_^1^, which satisfy the so called phase-matching condition:


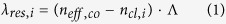


where *n*_*eff,co*_ and *n*_*cl,i*_ are the effective refractive index of the core and of the i-th cladding mode respectively, and Λ is the grating pitch. Nowadays, LPGs are very popular for sensing applications, due to their response to temperature and strain induced effects. Moreover, the intrinsic sensitivity to the material surrounding the cladding in the grating area, have made this devices very popular also as refractive index sensors^3^,^4^,^5^,^6^. For their fabrication, many techniques were studied, the most important using: UV radiations^1^,^7^, CO_2_ lasers^8^,^9^, infrared femtosecond lasers^10^, ion implantation^11^, mechanical deformations^12^, and electric arc discharge^13^,^14^,^15^.

In this work, we focus the attention on LPGs fabricated by the electric arc discharge (EAD) technique, where the perturbation is obtained by applying an electric arc to the optical fiber with a defined periodicity. The induced effect is twofold: (i) a localized tapering of the transversal size of the core and cladding regions along the fiber; (ii) a change in the silica refractive index. Different works focused on the mechanisms of grating formation, with different effects considered, as: fiber geometrical changes, silica stress relaxation, dopant diffusion and micro-deformations^13^,^14^,^16^,^17^. In principle, the fabrication of EAD-induced LPGs is possible in all kinds of optical fibers, and it was successfully demonstrated in boron co-doped fibers^18^, pure-silica core fibers^19^, photonic crystal fibers^20^,^21^, hollow core fibers^22^,^23^,^24^, cladding-etched fibers^25^,^26^. Moreover, the authors have recently successfully applied the technique to Fluorine-doped fibers^15^,^27^,^28^, which are fundamental for applications in radiation environments^29^,^30^,^31^, since they are significantly less sensitive to radiation as compared to standard Ge-doped fibers^19^,^32^.

Indeed, among numerous advantages, optical fiber sensors can withstand high radiation levels, leading to great interest in high radiation environments application, for example in spaceborne equipments or in nuclear installations, as well as in medical applications. High- and low-energy ionizing radiation defect generation in optical materials has been studied over several decades giving rise to excellent papers^33^,^34^,^35^. The most known radiation effect in optical fibers is the radiation-induced absorption (RIA), and it is recognized that the RIA may depend in a very complicated manner on the fiber chemical composition, radiation environment, and manufacturing process^35^. Radiation hard fibers, which are made of pure silica, for doses up to MGy level show only small radiation-induced effects. Differently, the doping with Ge, B, P, Al, etc. significantly increases the radiation sensitivity even for sub-mol% levels. For most of the applications operating in the near-IR and IR (above 1 μm), Ge-doped fibers can be used, the RIA levels remaining acceptable. However, such fibers are characterized by very high radiation-induced losses in the UV, and RIA in the visible domain is considerably higher than in pure-silica core fibers^34^,^35^,^36^,^37^.

Similarly, also Fiber Bragg gratings were broadly experimented under radiations, in order to assess their feasibility in nuclear environments as sensing devices. The results depend on the type of the optical fiber and the technology used for grating fabrication and thus there is not a standard classification of radiation sensitivity of FBGs, and its dependence on various parameters is not obvious^34^. About Long Period Gratings, there are only few reports concerning their behaviour under gamma ray irradiation. A detailed state-of-art concerning LPGs under gamma irradiation can be found in refs 30 and 31. Briefly, tests on LPGs inscribed by CO_2_ laser in N-doped optical fiber and by UV radiation in Ge-doped optical fiber are reported in ref. 32. Whereas in ref. 19, LPGs by electric arc discharge in pure-silica-core fibers with F-doped silica cladding, irradiated by gamma source, are presented. A LPG manufactured in F-doped fiber, being produced by CO_2_ method was studied in ref. 30. Some other works dealing with specially designed long period gratings report their sensitivity to gamma radiation. In ref. 36, chiral type LPGs from five different suppliers, were irradiated under gamma field. Whereas, in ref. 38 LPGs working in turning point region produced in B/Ge co-doped fiber by CO_2_ technique were investigated as well. It is important to remark here that the dependence of the radiation effects on fiber gratings is related to the induced changes of the effective refractive indices, of the core mode and core and cladding modes for FBGs and LPGs respectively. Even if there exists a relationship by the Kramers–Kronig relation, often no direct correlation between the induced absorption (RIA) and the refractive index change was found^34^,^39^.

In this work, we present a comparative experimental and theoretical study on the gamma radiation sensitivity of long period fiber gratings, fabricated by electric arc discharge technique, in different single mode fibers (SMF). In particular, we considered two Ge-doped fibers, following the standard Corning SMF28 provided by OZ Optics Ltd. and Thorlabs Inc., respectively, and the radiation resistant R1310-HTA fiber manufactured by Nufern Inc. To the best of our knowledge, this is the first time that LPGs achieved by arc discharge technique in standard fibers were studied on-line under gamma irradiation and compared with similar grating in radiation resistant fibers. The measurements were performed in real-time and a total dose of 35 kGy was reached, with an average dose rate of 0.18 kGy/h. Moreover, a study on the radiation-induced effects was conducted by using an experimental and numerical mixed approach. Finally, the radiation induced changes in the LPGs’ sensitivity to temperature and surrounding refractive index changes were also addressed.

## Results

### Gamma irradiation of Long Period Gratings

In this subsection, the effects of gamma radiation on Long Period Gratings are presented. In particular, for the purpose of this work, LPGs were fabricated by means of electric arc discharge technique in different optical fibers: two Ge-doped fibers according with Corning SMF28 provided by OZ Optics Ltd. and Thorlabs Inc., respectively, and the radiation resistant Nufern R1310-HTA fiber. Last fiber is declared by the manufacturer as optically and mechanically similar to SMF28, but with improved radiation resistance^40^,^41^. For intellectual property rights reasons, the manufacturers are usually not keen in providing exact information on the chemical composition of their fibers and the manufacturing technologies used, which make difficult a correlation of radiation effects to the optical fiber type.

These fibers were selected to: (i) investigate the effects of gamma radiation on LPG fabricated by EAD in standard Ge-doped fiber; and (ii) compare the radiation effects of LPGs in standard and radiation resistant fibers. Standard Ge-doped fibers were widely investigated in relation to radiation effect in terms of RIA and FBG shift^34^,^35^. Whereas few works report on the effect of CO_2_ based LPGs^31^,^38^ but there is no evidence of radiation effects on LPGs fabricated by EAD. Besides, the R1310-HTA fiber was investigated as it concerns the radiation effects in terms of RIA by the Nufern team^40^,^41^, but no gratings (FBGs and/or LPGs) were produced and irradiated so far in this type of fiber. They demonstrated that the RIA of R1310-HTA fiber is less than 50% of the Ge-doped standard fiber value over a wide dose range.

**Figure 1:**
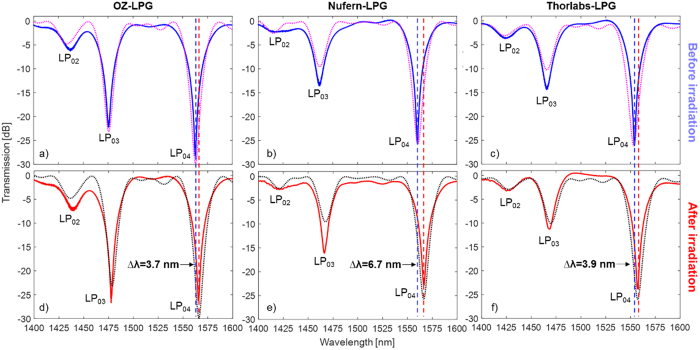
Full spectra of gratings: comparison between experimental (solid lines) and numerical results (dotted lines), before (blue and magenta lines) and after gamma irradiation (red and black lines) of 35 kGy.

The spectra of the three gratings are illustrated in Fig. 1(a,b,c), being identified respectively as OZ-LPG, Nufern-LPG, and Thorlabs-LPG depending on the hosting fiber. The fabrication period Λ of the three gratings was changed according to the hosting fiber, in order to tune the attenuation band related to the same cladding mode (LP_04_ in our case) around 1560 nm. Figure 1(a,b,c) also report the numerical LPGs’ spectra as dotted lines, obtained by coupled-mode theory (CMT) accordingly with approach proposed by Anemogiannis *et al*.^42^, and further investigated by Del Villar *et al*.^43^. This model allows determining the spectrum of a LPG, given the properties of the hosting fiber and grating, e.g.: fiber geometry, core and cladding dispersive refractive index, external medium refractive index, grating modulation strength, grating period, and grating length.

The irradiation of the three gratings was performed at room temperature, by placing the samples in the vicinity of a ^60^Co gamma source, and reaching a total dose of about 35 kGy at the end of the experiment (with an average dose rate of 0.18 kGy/h). A detailed description of the optical fibers, the LPGs’ fabrication parameters and procedure, as well as the irradiation and measurements setup, are reported in “Methods” section.

The transmitted spectra of the irradiated LPGs (i.e. after a total dose of 35 kGy) are reported in Fig. 1(d,e,f), in comparison with the original ones (shown respectively in Fig. 1(a,b,c)). As evident, all the attenuation bands exhibit a red shift that is significantly dependent on the fiber hosting the grating. Concerning the LP_04_ band, similar wavelength shifts of 3.7 nm and 3.9 nm were measured for the OZ-LPG and Thorlabs-LPG, respectively. Consequently we can state that the behaviour of the two Ge-doped fibers is very similar against the radiation effects. The difference of the 5% in the wavelength shifts of the resonance wavelengths of the LP_04_ cladding mode is partially due to the different period of the two gratings. Differently, the LP_04_ band of Nufern-LPG exhibits significantly higher red shift of about 6.7 nm.

Accordingly with considerations reported in previous papers^34^,^36^,^37^, it is expected that the exposure to gamma radiation induces an increase of the refractive index in the core region of the optical fiber. Moreover, this effect is confined in the core due to photosensitivity of dopants. Thus, the refractive index of core and cladding after the irradiation can be expressed as:


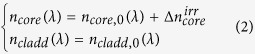


where *n*_*core,0*_*(λ)* and *n*_*cladd,0*_*(λ)* represent the core and cladding refractive index of the pristine fiber, respectively and 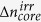 represents the radiation induced change of core refractive index. As one can observe from Fig. 1(d,e,f), for each grating the wavelength shifts of all bands (from LP_02_ to LP_04_) are in agreement with results from numerical simulations, where the dispersion of the core and cladding refractive index was considered and where it was considered 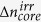 = 1.50 ∙ 10^−5^, 1.55 ∙ 10^−5^, and 2.3 ∙ 10^−5^ (related to a total dose of 35 kGy) for OZ, Thorlabs and Nufern fibers, respectively. Table 1 summarizes the wavelength shifts of all gratings.

**Figure 2:**
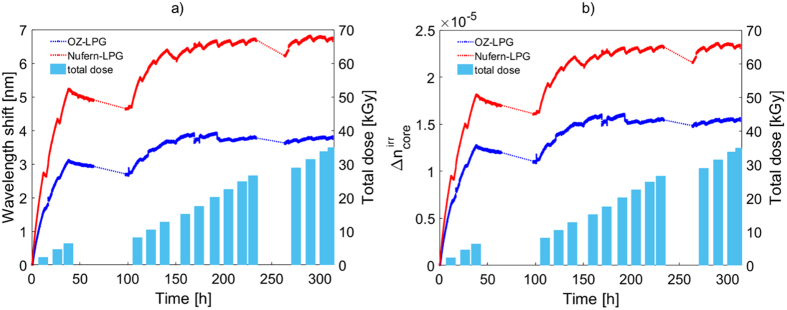
(**a**) Measured variation of the LP_04_ resonance wavelength with time and dose during the irradiation; (**b**) Radiation induced changes in the core RI of the fibers by OZ Optics and Nufern with time and dose, as obtained from numerical simulations.

A deeper study of the effect of the gamma radiation can be carried out by the real-time monitoring of resonant wavelength shift as function of the radiation exposure time. Figure 2(a) plots the shifts of the LP_04_ resonant wavelength of OZ-LPG and Nufern-LPG, as well as the accumulated dose plotted as bars. It should be remarked that Thorlabs-LPG was irradiated in the same condition of the other two gratings but, due to a limitation on the number of optical fibers connecting the irradiation chamber and the measuring unit, only OZ-LPG and Nufern-LPG could benefit from the real-time monitoring. The data acquired by the optical interrogator were thermally compensated by monitoring the temperature inside the box using a thermocouple and considering the thermal sensitivity of the gratings measured before the irradiation. Anyway, it should be highlighted that, during the irradiation, a maximum change in temperature of 1 °C occurred inside the insulated box containing the LPGs. Such change resulted in a wavelength shift for the gratings of about 50 pm, value which is two order of magnitudes lower than the radiation induced wavelength shifts. Concerning the strain effects, the gratings were fixed into a plastic frame, in order to keep their strain status unchanged during the irradiation.

As one can observe from Fig. 2(a), wavelength shift monotonically increases with dose increasing, experiencing higher rates at lower doses. In addition, a recovery behaviour is also visible, when the irradiation is paused. These irradiation breaks can be identified by looking at the gaps between the dose bars, and are due to the use of an industrial irradiator, where from time to time, the gamma source is retracted in the water tank in order to unload and reload products to be irradiated. Accordingly with spectral analysis, wavelength shifts of 3.7 nm and 6.7 nm were recorded, for OZ-LPG and Nufern-LPG, respectively, after a total dose of 35 kGy. Moreover, a saturation behaviour can be observed for both gratings, for doses higher than 15 kGy.

In refs 40 and 41 it was demonstrated that Nufern R1310-HTA fiber has a lower RIA with respect to standard SMF28 fiber, with a value of ~30 dB/km for a total dose of 10 kGy and dose rate of about 80 Gy/h. On the other hand, as reported in Fig. 2(a), Nufern-LPG experienced higher radiation sensitivities as compared with the gratings produced in standard fiber. According to equation (1), this means that the variation of the difference between effective refractive indices of the core and cladding modes in Nufern fiber is higher than in the standard fiber. With the hypothesis that radiation affects only the core region, the change in the Nufern core refractive index is higher than in the standard fiber. This behaviour can be explained by assuming that the ionizing radiation generates different defects in this fiber due to the doping level. Moreover, this result is not surprising since often no direct correlation was found between the induced absorption and the refractive index change^34^,^36^,^39^ and thus the label “radiation resistant fiber” is primarily related to a lower RIA.

In order to better understand the phenomena, for the first time to our knowledge, the measured wavelength shifts data during irradiation were combined with numerical simulations in order to quantify the core RI induced changes during the whole experiment. The results are reported in Fig. 2(b), with changes in the core RI found in the range 1.5–2.5 ∙ 10^−5^ after a total dose of 35 kGy, in good agreement with previous papers^31^,^32^,^37^,^44^.

**Figure 3:**
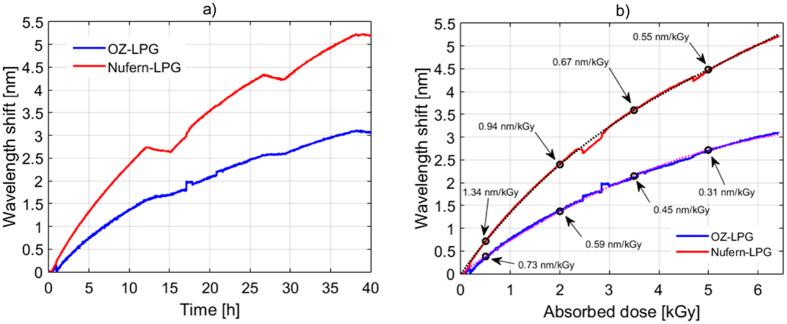
(**a**) Shifts in LP_04_ resonance wavelength during the first 40 hours for the two LPGs; (**b**) Measured shifts and sensitivity reported versus absorbed dose.

Moreover, in order to highlight the radiation response of LPGs, in Fig. 3(a) it is reported a zoom view of the wavelength shifts during the first forty hours of irradiation. For the same purpose, in Fig. 3(b) we represented the resonant wavelength shifts of both LPGs versus the absorbed dose (the radiation pauses being deleted). Here, a second order polynomial fitting was also added as dashed lines. It was found that the sensitivity of resonant wavelength to absorbed dose decreases with time, i.e. with dose, with sublinear behaviour. High sensitivities were recorded by both LPGs during the first twelve hours of irradiation, corresponding to doses up to 2.2 kGy. In particular, radiation sensitivities of 0.73 nm/kGy and 1.34 nm/kGy were recorded respectively by OZ-LPG and Nufern-LPG, for absorbed dose values of 0.5 kGy. Anyway, high sensitivities were recorded up to a total dose of 6.5 kGy. For example, at 2.0 kGy the sensitivities were 0.59 and 0.94 nm/kGy for OZ-LPG and Nufern-LPG respectively, whereas the same sensitivities at a dose of 5.0 kGy were 0.31 and 0.55 nm/kGy. The sensitivity found in the case of Nufern-LPG for doses up to 0.5 kGy, is comparable or even higher to the values of 1.0 and 1.2 nm/kGy reported in refs 31 and 36, respectively, establishing the basis for the development of a high sensitivity fiber optic radiation dosimeter. It is worth highlighting that, to this aim, further work is necessary to investigate the dependence of the LPG response upon the dose rate, post-recovery effects, and temperature dependence. However these topics lie beyond the scope of this paper. Until now there are few attempts concerning a systematic investigation in respect to dose rate change over several orders of magnitude, because of high irradiation costs. For example, in ref. 36 it is reported that the ratio of the wavelength shifts of chiral LPGs at only two dose rates (differing by one order of magnitude) was in the range 1.1–1.2.

### Effect of gamma radiation on LPGs’ sensing features

This subsection compares the sensing features of LPGs to surrounding refractive index and local temperature before and after the irradiation process.

#### Sensitivity to external refractive index

**Figure 4:**
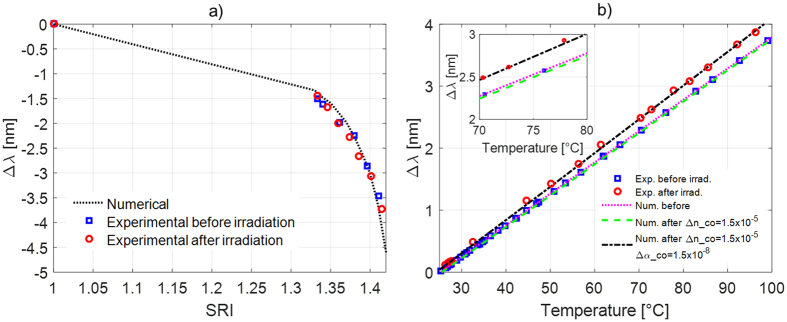
(**a**) Relative wavelength shift of LP_04_ in OZ-LPG induced by SRI changes: comparison between experimental values before (blue squares) and after irradiation (red circles), and numerical simulations results (dotted line); (**b**) Temperature induced wavelength shifts in OZ-LPG, comparison between experimental values before (blue squares) and after irradiation (red circles), together with numerical simulations results for different cases. In inset, a zoom over the region 70–80 °C is given to highlight the differences.

For the characterization of LPGs sensitivity to variations in the surrounding refractive index (SRI), the gratings were immersed in different solutions with known refractive index, in the range 1.33–1.42, in order to evaluate the wavelength shift of attenuation bands, before and after the irradiation. More details about the setup can be found elsewhere^5^,^15^. In Fig. 4(a) it is plotted the LP_04_ resonant wavelength shifts (with respect to the value in air) for OZ-LPG. According to the general LPG behaviour, attenuation bands exhibit a blue shift with SRI increasing^3^.

As one can observe, after the irradiation, small changes were found but they can be considered within the measuring error. Moreover, in the same figure the experimental values are compared to numerical simulation results, being not reported for post irradiation situation because they practically overlap with those before irradiation. A good agreement can be observed between experimental data and numerical values, confirming both the performances and repeatability of the LPG fabrication technique and the accuracy of the numerical model. The trivial changes found in SRI sensitivity are in agreement with the fact that gamma radiations only affected the core region, while the effect was negligible for the cladding. This means that the effective refractive index of the cladding modes, which are the responsible of SRI sensitivity (according to equation (1)), are not modified. Same considerations hold for the other gratings as well.

#### Sensitivity to temperature

The temperature characterization was performed by using a chamber similar to the one described in ref. 15, the temperature monitoring was done using a commercial FBG-based sensor and the temperature range investigated was 25–100 °C.

To understand the effect of radiation on LPGs temperature sensitivity, Fig. 4(b) plots the LP_04_ wavelength shifts (with respect to that at 25 °C) for OZ-LPG before and after irradiation with blue square and red circle markers, respectively. In both cases, the attenuation band exhibits a red shift with the temperature increase, and following a linear behavior^3^. However, the sensitivity of LP_04_ increases from the value of 50.5 pm/°C before irradiation to 53.8 pm/°C after irradiation. An increase in temperature sensitivity was found for the other gratings as well. In particular, for Nufern-LPG, the sensitivity of LP_04_ changed from 49.3 to 49.6 pm/°C, whereas in Thorlabs-LPG, the change in sensitivity was from 49.1 to 51.5 pm/°C for the same cladding mode.

For the understanding of the radiation effects on the LPG’s temperature sensitivity, theoretical and numerical analysis were carried out. The temperature sensitivity of a cladding mode can be derived from equation (1), by applying the partial derivative in respect to temperature:


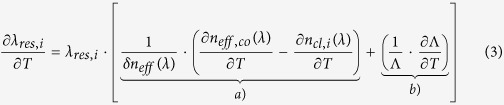


where *δn*_*eff*_, is the difference between the effective refractive index of the core mode and cladding mode. The term (b) is related to the silica thermal expansion and can be usually neglected since it is some orders of magnitude smaller than the term (a), which is referred to as the thermo-optic effect. Such contribution depends on the product of the inverse of the difference between the effective refractive index of core and cladding, and the difference between the thermal sensitivities of the same indices.

In order to consider this effect, numerical simulations were carried out by considering the dependence of the core and cladding refractive index on the temperature changes as:


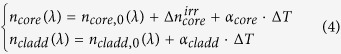


where *α*_*core*_ and *α*_*cladd*_ are the thermal coefficients of the core and cladding respectively, and ∆*T* is the temperature change. For the standard OZ fiber *α*_*core*_ and *α*_*cladd*_ are fixed to 7.97 ∙ 10^−6^/°C and 7.80 ∙ 10^−6^/°C respectively, accordingly with refs 3 and 45 whereas the refractive index increase related to radiation was zero, 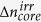 = 0, in the situation before irradiation. The result is also reported in Fig. 4(b) (as magenta dotted line) showing very good agreement with the experimental ones.

Concerning the irradiated gratings, the figure Fig. 4(b) also illustrates the numerical derived thermal response of the grating (green dashed line) considering the value 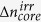 = 1.50 ∙ 10^−5^, according to the value reported in earlier, while *α*_*core*_ and *α*_*cladd*_ were kept unchanged. However, differently from the experimental results, this leads to a small decrease (−0.4 pm/°C) in the temperature sensitivity of the cladding mode. In fact, such change, as shown in equation (4), acts by increasing *δn*_*eff*_ and hence lowering the sensitivity.

As matter of fact, to fit the experimental data a change in the core thermo-optic coefficient, *α*_*core*_, needs to be considered, whereas the *α*_*cladd*_ is kept unchanged accordingly with the assumptions in previous section. A good agreement between numerical and experimental thermal sensitivities of post-radiated LPG was achieved, considering a change in the core thermo-optic coefficient, *α*_*core*_, of 1.5 ∙ 10^−8^/°C. Similar results hold for the other two gratings, with changes in the core thermo-optic coefficient of 1.0 ∙ 10^−9^/°C and 1.0 ∙ 10^−8^/°C for Nufern and Thorlabs fiber respectively.

## Discussion

In this work, we studied as a premiere, experimentally and theoretically, gamma radiation sensitivity of long period gratings, fabricated by electric arc discharge technique, in different SMF fibers supplied by OZ Optics Ltd., Nufern Inc., and Thorlabs Inc.

The measurements were performed in real-time during the irradiation process, up to a total dose of 35 kGy, with an average dose rate of 0.18 kGy/h. It is the first time that LPGs written in standard fibers by electric arc discharge are tested on-line under gamma irradiation and compared with LPG in radiation resistant fiber written by the same technique. The investigation on the radiation-induced effects was conducted by combining both experimental and numerical results. The numerical model for the simulation of LPGs’ full spectra was developed based on the coupled mode theory approach and a good agreement was reached with the experimental results.

During the irradiation process, all the attenuation bands of the three LPGs exhibited a red shift, significantly dependent on the type of the hosting fiber. The former effect is a consequence of the increase in the core refractive index of the optical fiber, due to the radiation induced generation of dopant-related defects^34^,^36^,^37^. In particular, concerning the LP_04_ band, similar wavelength shifts of 3.7 nm and 3.9 nm were measured for the OZ-LPG and Thorlabs-LPG, respectively, after a total dose of 35 kGy. Both fibers are declared to meet the standard Corning SMF28 and the LPG fabrication period was slightly different. Differently, the LP_04_ band of Nufern-LPG exhibited a significantly higher red shift of about 6.7 nm after exposure to the same dose. Concerning the radiation sensitivities, values of 0.73 nm/kGy and 1.34 nm/kGy were recorded respectively by OZ-LPG and Nufern-LPG, for absorbed dose values of 0.5 kGy. The sensitivity of Nufern-LPG can be placed among the highest values reported in literature^31^,^36^.

The different behaviour of these fibers could be justified by a different doping concentration^37^, as from the different NA aperture of the Nufern R1310-HTA fiber respect to the standard SMF28. This fiber is declared by the manufacturer and demonstrated to be radiation tolerant^40^,^41^, i.e. having a lower RIA in respect to SMF28. It should be remarked that RIA and LPG wavelength shifts are not always in agreement, since the former is related to the absorption of the core mode whereas the latter is related to the difference between core and cladding mode effective indices^34^,^36^,^39^.

By combining experimental data and results from accurate numerical model, the gamma radiation induced change of the core refractive index, i.e. 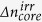, was also found to be 1.50 ∙ 10^−5^, 1.55 ∙ 10^−5^, and 2.3 ∙ 10^−5^ for OZ, Thorlabs and Nufern fibers, respectively. Moreover, the influence of the irradiation process on the LPGs’ sensing features, in respect to external refractive index and temperature changes were also investigated. In particular, after the irradiation an increase in thermal sensitivity was measured in all three gratings, with different magnitude depending on the hosting fiber. It was found that, considering the aforementioned change in the core refractive index, was not enough to justify such behaviour. In fact, the increase of the temperature sensitivity could be only attributed to changes also in the core thermo-optic coefficient, *α*_*core,*_ considering values of 1.5 ∙ 10^−8^/°C, 1.0 ∙ 10^−8^/°C, and 1.0 ∙ 10^−9^/°C for OZ, Thorlabs and Nufern fibers respectively. Concerning the SRI sensitivity, no appreciable changes were found after the irradiation, proving that the effect of radiation is negligible on the pure silica cladding region for such fibers.

## Methods

### Long Period Gratings fabrication by electric arc discharge

All three LPGs considered in this work were fabricated by means of the electric arc discharge technique. This procedure requires that a certain length of uncoated optical fiber is positioned between two electrodes, in order to apply the arc discharge to the fiber. One end of the fiber is moved by means of a translation stage, while the other one is kept under constant axial tension. Finally, a sequence of arc discharges and fiber displacement is repeated several times until the desired LPG’s spectral features are reached^14^. A detailed report of the fabrication setup used for this work is reported elsewhere^5^,^15^. Briefly, arc discharges were provided by the electrodes of a Sumitomo Type-39 fusion splicer. Typical range for the discharge parameters are: the power in range of 1–15 step (proprietary unit from manufacturer) and duration of 200–900 ms. A constant force was applied to the fiber by using a 12 g weight. The fiber displacement was operated by using a micro-stepper (with resolution better than 1 μm). Finally, during the fabrication the gratings transmission spectra were recorded with OSA model Yokogawa AQ6370B (resolution set to 0.1 nm), while the illumination was provided by a broadband source (involving several SLEDs in range 1100–1700 nm).

For the purpose of this work, the gratings were fabricated in different optical fibers:

- two Ge-doped SMF28 fiber having Dcore = 8.2 μm, Dclad = 125 μm, MFD = 10.4 ± 0.8 μm @ 1550 nm, and NA = 0.14 supplied by OZ Optics Ltd. and Thorlabs Inc, respectively;
- R1310-HTA fiber manufactured by Nufern Inc., with Dcore = 9.0 μm, Dclad = 125 μm, MFD = 10.5 ± 1.0 μm @ 1550 nm, and NA = 0.12. This fiber is declared by the manufacturer as optically and mechanically similar to SMF28 but with improved radiation performances^40^,^41^;

All gratings were produced by using arc power in range 1–5 step and arc durations of 400–450 ms, resulting in devices with final length shorter than 25 mm. The spectra of the three gratings are illustrated in Fig. 1(a,b,c), being fabricated in OZ, Nufern, and Thorlabs fiber respectively. The period of the three gratings was changed depending on the hosting fiber, to tune the attenuation band related to the same cladding mode (LP_04_) around 1560 nm. Following details of LPGs are highlighted:

- OZ-LPG, was fabricated with period Λ = 628 μm. In the wavelength range 1400–1600 nm, three attenuation bands are visible, centred at 1437.0 nm (LP_02_), 1475.3 nm (LP_03_), and 1562.8 nm (LP_04_), with depth of 6.3 dB, 22.3 dB, and 28.3 dB, respectively.
- Nufern-LPG, having period Λ = 677 μm. Also in this case, there are three attenuation bands, centred at 1418.3 nm (LP_02_), 1461.8 nm (LP_03_), and 1560.2 nm (LP_04_), with depth of 2.4 dB, 13.7 dB, and 25.6 dB, respectively;
- Thorlabs-LPG, with Λ = 646 μm. In the spectrum, three attenuation bands are visible, centred at 1423.8 nm (LP_02_), 1465.4 nm (LP_03_), and 1554.0 nm (LP_04_), with depth of 3.8 dB, 14.5 dB, and 26.1 dB, respectively.

Note that even if two Ge-doped fibers are (in principle) similar, the fiber fabrication tolerance makes same differences. Indeed, the LPG fabrication demands a slightly different period in order to achieve LP_04_ cladding mode tuned at same spectral position.

### Irradiation and measurement setup

The irradiation was performed at room temperature, a total dose of about 35 kGy was reached at the end of the experiment, as a result of about 194 hours of irradiation (total experiment was 322 hours long) with an average dose rate of 0.18 kGy/h. During the irradiation process the LPGs samples, each one fixed into a plastic frame to avoid strain induced effects, were placed into a thermally insulating box in the vicinity of the ^60^Co gamma source. This gamma source produced by the Institute of Isotopes Co. Ltd. in Budapest is a class IV (source storage in a pool, automatic transport system) in operation at the IRASM accredited facility of the “Horia Hulubei” National Institute for Physics and Nuclear Engineering, Magurele, Romania. The dose measurements were performed with an Alanine-EPR dosimetric system, by placing the dose monitor close to the samples. The dosimetry system is traceable at National Physics Laboratory (UK) via Risoe High Dose Reference Laboratory (Denmark). The uncertainty of the dose/dose rate is 3.3% at one standard deviation. In order to monitor the temperature changes during the exposure to radiation, two thermocouples were used, one located inside of the box carrying the samples and one placed outside this box, to monitor the ambient temperature in the irradiation facility. The two temperature sensors were connected to the inputs of a NI-cRIO-9211 temperature data logger with USB connection. The temperature measurement results were employed to perform the appropriate corrections of LPGs wavelength shift detected during the irradiation.

**Figure 5:**
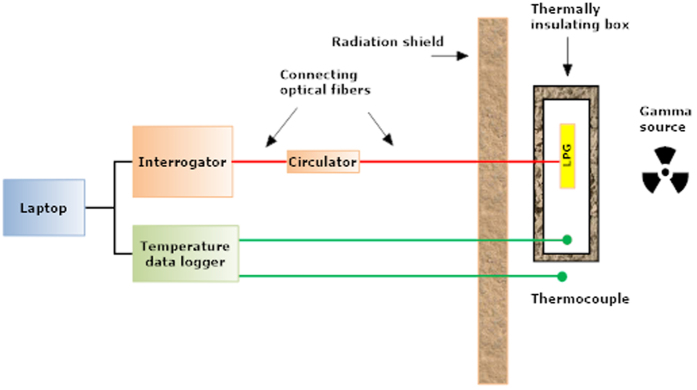
The sketch of irradiation and measurement setup (not at scale).

The experiment was controlled and data were collected remotely by a laptop, placed at a distance of about 20 m away from the ^60^Co source. For shielding reasons all electrical wires and connecting optical fibers were positioned along a labyrinth-like path. A schematic view of the irradiation setup is shown in Fig. 5 where the three LPGs were irradiated simultaneously. For real-time monitoring of resonant wavelength shift as function of the radiation exposure, LPGs spectra were acquired by the sm125 Micron Optics interrogator with scanning time of thirty-seconds. The interrogator has a wavelength accuracy of 1 pm and a repeatability of 0.2 pm (according to the definitions reported in NIST Technical Note 1297). The interrogator was operated in the transmission mode by means of an optical circulator for each grating. However, the operating wavelength range of sm125 (1510–1590 nm) permits a real-time monitoring only of the attenuation band related to LP_04_ cladding mode. Moreover, since we had a limited number of fibers connecting the radiated region (LPGs chamber) and the control unit, only two LPGs (OZ-LPG and Nufern-LPG) could benefit from the real-time monitoring.

The optical fibers connecting the instrumentation to LPG samples were shielded during the irradiation, to avoid additional degradation of the optical transmission S/N, which might be produced by their attenuation increase. As the irradiation facility is an industrial irradiator LPGs exposure to gamma-rays was not continuous, the ^60^Co source being stored from time-to-time in the water pool for the change of the goods subjected to irradiation.

## Additional Information

**How to cite this article:** Esposito, F. *et al*. Real-time analysis of arc-induced Long Period Gratings under gamma irradiation. *Sci. Rep.* **7**, 43389; doi: 10.1038/srep43389 (2017).

**Publisher's note:** Springer Nature remains neutral with regard to jurisdictional claims in published maps and institutional affiliations.

## Figures and Tables

**Table 1:** LPGs wavelength shifts after irradiation.

ID	∆λ LP_02_ [nm]	∆λ LP_03_ [nm]	∆λ LP_04_ [nm]
OZ-LPG	3.1	3.2	3.7
Nufern-LPG	5.5	5.7	6.7
Thorlabs-LPG	3.2	3.4	3.9
